# Molecular epidemiology of livestock rabies viruses isolated in the northeastern Brazilian states of Paraíba and Pernambuco from 2003 - 2009

**DOI:** 10.1186/1756-0500-5-32

**Published:** 2012-01-16

**Authors:** Nobuyuki Mochizuki, Hiroyuki Kawasaki, Maria LCR Silva, José AB Afonso, Takuya Itou, Fumio H Ito, Takeo Sakai

**Affiliations:** 1 Nihon University Veterinary Research Center, Nihon University, 1866 Kameino, Fujisawa, Kanagawa 252-0880, Japan; 2 Academic Unit of Veterinary Medicine, Center of Health and Rural Technology, Federal University of Campina Grande, Campus of Patos, Avenida Santa Cecília, P.O. Box 64, 587000-000 Patos, PB, Brazil; 3 Bovine Clinic, Federal Rural University of Pernambuco, Campus of Garanhuns, Avenida Bom Pastor s/n, Bairro Mundaú, Bairro Mundaú Boa Vista, SP.O. Box 152, 55292-270 Garanhuns, PE, Brazil; 4 Department of Preventive Veterinary Medicine and Animal health, Faculty of Veterinary Medicine and Zootechny, University of São Paulo, Av. Prof. Dr. Orlando Marques de paiva, 87, Cidade Universtiátria, São Paulo 05508-000, SP, Brazil

## Abstract

**Background:**

Limited or no epidemiological information has been reported for rabies viruses (RABVs) isolated from livestock in the northeastern Brazilian states of Paraíba (PB) and Pernambuco (PE). The aim of this study was to clarify the molecular epidemiology of RABVs circulating in livestock, especially cattle, in these areas between 2003 and 2009.

**Findings:**

Phylogenetic analysis based on 890 nt of the nucleoprotein (N) gene revealed that the 52 livestock-derived RABV isolates characterized here belonged to a single lineage. These isolates clustered with a vampire bat-related RABV lineage previously identified in other states in Brazil; within PB and PE, this lineage was divided between the previously characterized main lineage and a novel sub-lineage.

**Conclusions:**

The occurrences of livestock rabies in PB and PE originated from vampire bat RABVs, and the causative RABV lineage has been circulating in this area of northeastern Brazil for at least 7 years. This distribution pattern may correlate to that of a vampire bat population isolated by geographic barriers.

## Background

Rabies is a fatal infectious disease that causes encephalomyelitis. In Brazil, various rabies viruses (RABVs) have been isolated from numerous animal species, including dogs, foxes, cats, and cattle, as well as from hematophagous, insectivorous, and frugivorous bats. Vampire bats, particularly *Desmodus rotundus*, are an important rabies vector in Latin America. Transmission from vampire bats to humans has been reported, primarily in the Amazon regions of Brazil and Peru, and a large number of cases of cattle rabies transmitted by vampire bats also have been reported in Brazil [1]. Since the introduction of a regional elimination program, the incidence of human and canine rabies in Latin America has fallen by 90% over the past 20 years. However, northeastern Brazil remains a "hotspot" for human rabies because of circulation of the virus among the dog population [2]. Carnieli Jr. *et al. *reported the molecular characterization and epidemiology of RABVs isolated from canids in northeastern Brazil [3-5], and Shoji *et al. *reported the genetic and phylogenetic characterization of RABVs isolated from wild fox, insectivorous bats, and livestock in Paraíba (PB) [6]. In Olinda, a city in Pernambuco (PE), 7,062 patients underwent prophylactic antirabies treatment between 2002 and 2006 [7]. Molecular and geographic analyses of livestock rabies in central and southeast Brazil revealed that RABVs isolated from livestock were related to the virus found in vampire bat populations, and this epidemiological pattern was maintained over time and space in these areas [8-11]. However, little or no information on the molecular epidemiology of RABVs has been reported for the virus isolated from livestock in PB and PE. The aim of the present study was to analyse the molecular epidemiology of RABVs circulating among livestock, especially cattle, in these areas between 2003 and 2009.

## Results

The sequences of 890 nt PCR products, corresponding to nucleotides 89-978 of the Pasteur vaccine (PV) strain, were determined for all 52 RABV isolates. Among the 52 isolates, the nucleotide and amino acid sequence identities were 97.7-100% and 97.9-100%, respectively.

**Figure 1:**
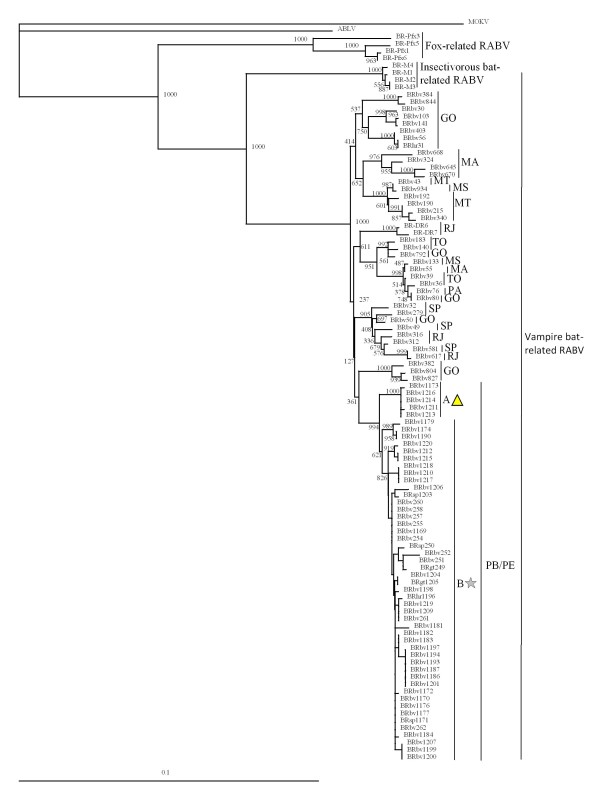
**A phylogenetic tree based on the nucleotide sequences of the N gene**. A phylogenetic tree based on the nucleotide sequences of 890 nt (bases 89-978) of the N gene was constructed using the method devised by Saitou and Nei [12]; the bootstrap probabilities of each node were calculated using 1,000 replicates. The designations BRbv, BRsp, BRgt, BRhr, BR-Pfx, and BR-DR indicate samples from Brazilian cattle, sheep, goats, horses, foxes, and vampire bats, respectively. MOKV and ABLV denote the Mokola virus and Australian bat lyssavirus, respectively. State abbreviations are as follows: PB, Paraíba; PE, Pernambuco; GO, Goiás; SP, São Paulo; RJ, Rio de Janeiro; MT, Mato Grosso; TO, Tocantins; MA, Maranhão; PA, Pará; MS, Mato Grosso do Sul. The triangle and star symbols represent the new sub-lineage (group A) and the previously reported lineage (group B), respectively.

**Figure 2:**
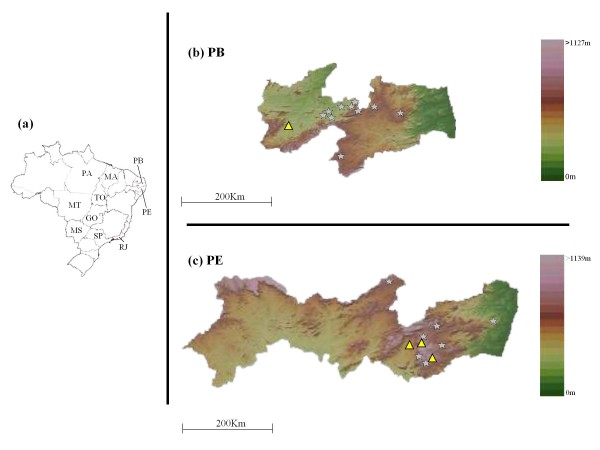
**Geographic distribution of rabies virus isolates in the Brazilian states of Paraíba and Pernambuco**. (**a**) Map of Brazil indicating location of states pertinent to this study. State abbreviations are as follows: PB, Paraíba; PE Pernambuco; GO, Goiás; SP, São Paulo; RJ, Rio de Janeiro; MT, Mato Grosso; TO, Tocantins; MA, Maranhão; PA, Pará; MS, Mato Grosso do Sul. (**b**, **c**) Detailed geographic distribution of livestock isolates classified as genetic variants in the states of PB (**b**) and PE (**c**). The two symbols (triangle and star) correspond to the new sub-lineage and previously reported lineage, respectively, as used in Figure 1. Samples for which the geographic origin and the genetic variant are identical are illustrated using the same symbol. Brazilian maps were obtained from Brasil em Relevo - Embrapa Monitoramento por Satélite http://www.relevobr.cnpm.embrapa.br/.

Phylogenetic analysis based on the sequences of 890 nt of the N gene revealed that the 52 RABV isolates included in this study clustered with a vampire bat-related RABV lineage; these isolates did not cluster with the dog-, fox-, or insectivorous bat-related RABVs (Figure 1). Comparison with RABVs isolated from other states in Brazil indicated that these 52 RABV isolates belonged to a single lineage; furthermore, this lineage was divided between a previously characterized main lineage (B) and a novel sub-lineage (A) consisting of several isolates located in PE. Geographical plotting showed that RABV isolates of the novel sub-lineage were derived from neighbouring areas (Figure 2). The topographical distributions of isolates from lineages A and B could not be distinguished in the areas covered by this study.

## Discussion

Segments of the N gene from the 52 RABV isolates, collected from PB and PE between 2003 and 2009, were sequenced and phylogenetically analysed. This N gene segment displayed greater than 97.7% nucleotide and amino acid sequence identity among these 52 RABVs. This correlation implies that the lineage has been maintained during transmission in PB and PE.

The phylogenetic analysis described here indicates that all of the livestock RABVs in PB and PE were derived from vampire bat rabies. Furthermore, the present study reveals that there are two RABV lineages that are separate from other regional Brazilian vampire bat-related RABV lineages. The first, sub-lineage A, consists of BRbv1173 (collected in PB in 2004) and another seven RABV isolates collected in PE in 2008-2009; the second, sub-lineage B, consists of isolates collected in PB in 2003-2009 and in PE in 2007-2009. Comparison among RABV isolates from multiple northern states of Brazil revealed that lineages from PB and PE were distinct from those obtained from the Maranhão (MA) state. Because of geographical barriers (mountains and rivers) between PB/PE and MA, it would be difficult for vampire bats (the prime vector for RABV) to move freely between PB/PE and MA. Geographical mapping (Figure 2) demonstrates that sub-lineage A was located mainly in high-altitude areas of PE, while sub-lineage B was widely distributed and present in both PE and PB. Thus, these two lineages seem to correlate with geographic factors and/or vampire bat populations, but do not appear to correlate with the year of isolation. Using a 203 nt segment (bases 109 to 311) of the N gene, Kobayashi *et al. *[8,9] reported the existence of at least 24 RABV genetic variants among vampire bat-transmitted cases of rabies in cattle in Brazil; the distribution of several of these RABV genetic variants was found to be delimited by geographic boundaries, including mountain ranges and rivers. Our analysis (data not shown) using the same 203 nt segment reveals that the 52 RABV isolates (from PB and PE) of the present study correlate with the same PB lineage described by Kobayashi *et al. *These results indicate that livestock rabies has been transmitted by vampire bats in PB and PE during this study period. Furthermore, this RABV lineage seems to have been circulating in this area for at least 7 years, with transmission affected by geographic factors and resulting in dispersion of vampire bats among regional populations.

Previous reports have shown human exposure to vampire bat-transmitted rabies in northeastern Brazil, including PE [1,7]. The present phylogenetic analysis suggests that rabies epidemics that occurred in cattle in PB and PE were transmitted by vampire bats. These RABV isolates comprised a lineage independent from that of other Brazilian isolates, with the distinction reflecting isolation from neighbouring regions by geographic factors. Thus, the vampire bat-derived rabies in this area represents an endemic disease, suggesting that the regional control of vampire bat rabies in this area may be a workable model for local elimination of human and livestock rabies.

## Conclusions

The present study indicates that occurrences of livestock rabies in PB and PE were caused by vampire bat RABVs, and that this RABV lineage has been circulating in this area of northeastern Brazil for at least 7 years. This pattern of distribution may correlate to that of a vampire bat population isolated by geographic barriers.

## Methods

The 52 RABV isolates used in this study were obtained from cattle (46), sheep (3), goats (2), and horse (1), and were collected in PB and PE between 2003 and 2009 (Table 1). Brain specimens from these livestock were diagnosed as RABV-positive by an immunofluorescent antibody test and a mouse inoculation test. These study procedures were implemented in accordance with the Institutional Guidelines for Animal Experiments at the Campina Grande University under the permission (number 129/2009) of the Committee for Experimental Animals of this College. Viral RNA was extracted from the brains of livestock using the QIAamp Viral RNA Mini Kit (Qiagen, Hilden, Germany). Nucleoprotein (N) gene sequences from the Brazilian RABV isolates were amplified using RT-PCR with primers JW12 (5'-ATGTAACACCYCTACAATG-3') (position: 55-73 of PV) and N8 (5'-AGTTTCTTCAGCCATCTC-3') (position: 1585-1568 of PV), followed by hemi-nested PCR with primer pairs as follows. Primer pair A consisted of JW12 and P2 (5'-CCCATATAACATCCAACAAAGTG-3') (position: 1029-1007 of PV), and generated a 975-nt amplicon. Primer pair B consisted of P1 (5'-CTACAATGGATGCCGACAAGA-3') (position: 66-86 of PV) and N8, and generated a 1,520 nt amplicon. The nucleotide sequences of RABVs from Brazilian foxes, livestock, and hematophagous and insectivorous bats were obtained from GenBank (Table 1). Cycle sequencing, nucleotide and amino acid sequence alignments, and phylogenetic analyses were performed as previously described [13,14]. The geographic origins of the RABV isolates sequenced from the Brazilian livestock were plotted at the municipal level of the respective federal states using MapInfo Professional GIS software (ver. 8.0, MapInfo Japan K.K., Tokyo, Japan). Brazilian maps were obtained from Brasil em Relevo - Embrapa Monitoramento por Satélite [15].

**Table 1:** Brazilian rabies virus isolates used in this study

Sample*1	Species	Location	State*2	Year	Lineage of PB and PE*3	**Accession No**.	Reference
BR-DR6	*Desmodus rotundus*	Laje de Muriae	RJ	1998		AB297633	[16]

BR-DR7	*Desmodus rotundus*	Itaperuna	RJ	1997		AB297634	[16]

BR-M1(BR-Pbt1)	*Molossus *sp.	Patos	PB			AB206414	[6]

BR-M2(BR-Pbt2)	*Molossus *sp.	Patos	PB			AB206415	[6]

BR-M3(BR-Pbt3)	*Molossus *sp.	Patos	PB			AB206416	[6]

BR-M4(BR-Pbt4)	*Molossus *sp.	Patos	PB			AB206417	[6]

BR-Pfx1	Fox	Patos	PB	2002		AB362483	[13]

BR-Pfx3	Fox	Patos	PB	2001		AB206409	[6]

BR-Pfx5	Fox	Patos	PB	2002		AB206411	[6]

BR-Pfx6	Fox	Patos	PB	2002		AB207884	[6]

BRbv30	Cattle	Morrinhos	GO	1999		AB083803	[17]

BRbv32	Cattle	Sao Roque	SP	1994		AB083805	[17]

BRbv36	Cattle	Nova Olinda	TO	1998		AB083809	[17]

BRbv39	Cattle	Colinas	TO	1999		AB083811	[17]

BRbv43	Cattle	Alto Taquari	MT	1999		AB083813	[17]

BRbv49	Cattle	Piraju	SP	1989		AB083817	[17]

BRbv50	Cattle	Corumbaiba	GO	1999		AB083818	[17]

BRbv55	Cattle	Montes Altos	MA	1998		AB675602*4	[8]

BRbv56	Cattle	Iporá	GO	1998		AB675603*4	[8]

BRbv76	Cattle	Xinguará	PA	2002		AB675604*4	[8]

BRbv80	Cattle	Ipameri	GO	2001		AB675605*4	[8]

BRbv103	Cattle	Nova Crixás	GO	2001		AB675606*4	[8]

BRbv133	Cattle	Xambioa	TO	2000		AB675607*4	[8]

BRbv140	Cattle	Natividade	TO	2000		AB675608*4	[8]

BRbv141	Cattle	Nova Crixas	GO	2000		AB675609*4	[8]

BRbv183	Cattle	Natividade	TO	2001		AB675610*4	[8]

BRbv190	Cattle	Pocone	MT	2002		AB675611*4	[9]

BRbv192	Cattle	Nobres	MT	2002		AB675612*4	[8]

BRbv215	Cattle	Rosario Oeste	MT	2002		AB675613*4	[9]

BRbv251(BR-Pbv1)	Cattle	Patos	PB	2003	PB/PE-B	AB206423	[6]

BRbv252(BR-Pbv2)	Cattle	Patos	PB	2003	PB/PE-B	AB206424	[6]

BRbv254(BR-Pbv3)	Cattle	Patos	PB	2003	PB/PE-B	AB206425	[6]

BRbv255(BR-Pbv5)	Cattle	Patos	PB	2003	PB/PE-B	AB206426	[6]

BRbv257(BR-Pbv7)	Cattle	Patos	PB	2003	PB/PE-B	AB206427	[6]

BRbv258(BR-Pbv8)	Cattle	Patos	PB	2003	PB/PE-B	AB206428	[6]

BRbv260(BR-Pbv10)	Cattle	Patos	PB	2003	PB/PE-B	AB206429	[6]

BRbv261(BR-Pbv11)	Cattle	Patos	PB	2003	PB/PE-B	AB206430	[6]

BRbv262(BR-Pbv12)	Cattle	Patos	PB	2003	PB/PE-B	AB206431	[6]

BRbv279	Cattle	Pirapozinho	SP	2002		AB675614*4	[9]

BRbv312	Cattle	Paulo de Frontin	RJ	1987		AB675615	This study

BRbv316	Cattle	Miguel Pereina	RJ	2000		AB675616*4	[9]

BRbv324	Cattle	Itapecuru Mirim	MA	2004		AB675617*4	[9]

BRbv340	Cattle	Nossa Senhora do Livramento	MT	2004		AB675618*4	[9]

BRbv382	Cattle	Orizona	GO	2002		AB675619*4	[9]

BRbv384	Cattle	Nova América	GO	2002		AB675620*4	[9]

BRbv403	Cattle	Piranhas	GO	2002		AB675621*4	[9]

BRbv581	Cattle	Tambaú	SP	2003		AB675622*4	[9]

BRbv617	Cattle	Rio Claro	RJ	2004		AB675623*4	[9]

BRbv645	Cattle	Capinzal do Norte	MA	2004		AB675624*4	[9]

BRbv668	Cattle	Santo Antônio dos Lopes	MA	2005		AB675625*4	[9]

BRbv670	Cattle	Godofredo Viana	MA	2005		AB675626*4	[9]

BRbv792	Cattle	Morrinhos	GO	2002		AB675627*4	[9]

BRbv804	Cattle	Pilar de Goiás	GO	2005		AB675628*4	[9]

BRbv827	Cattle	Cocalzinho de Goiás	GO	2005		AB675629*4	[9]

BRbv844	Cattle	Itapaci	GO	2006		AB675630*4	[9]

BRbv934	Cattle	Bandeirantes	MS	2005		AB675631*4	[9]

BRbv1169	Cattle	Patos	PB	2004	PB/PE-B	AB623080	This study

BRbv1170	Cattle	Santa Terezinha	PB	2004	PB/PE-B	AB623081	This study

BRbv1172	Cattle	São José do Bonfim	PB	2004	PB/PE-B	AB623082	This study

BRbv1173	Cattle	Itaporanga	PB	2004	PB/PE-A	AB623083	This study

BRbv1174	Cattle	São Vicente do Seridó	PB	2004	PB/PE-B	AB623084	This study

BRbv1176	Cattle	Patos	PB	2005	PB/PE-B	AB623085	This study

BRbv1177	Cattle	Santa Luzia	PB	2005	PB/PE-B	AB623086	This study

BRbv1179	Cattle	Areal	PB	2006	PB/PE-B	AB623087	This study

BRbv1181	Cattle	Monteiro	PB	2006	PB/PE-B	AB623089	This study

BRbv1182	Cattle	Junco do Seridó	PB	2006	PB/PE-B	AB623090	This study

BRbv1183	Cattle	São José do Sabugi	PB	2006	PB/PE-B	AB623091	This study

BRbv1184	Cattle	Santa Luzia	PB	2006	PB/PE-B	AB623092	This study

BRbv1186	Cattle	Patos	PB	2007	PB/PE-B	AB623093	This study

BRbv1187	Cattle	Patos	PB	2007	PB/PE-B	AB623094	This study

BRbv1190	Cattle	Areal	PB	2007	PB/PE-B	AB623096	This study

BRbv1193	Cattle	Patos	PB	2007	PB/PE-B	AB623097	This study

BRbv1194	Cattle	São José do Bonfim	PB	2007	PB/PE-B	AB623098	This study

BRbv1197	Cattle	São José do Bonfim	PB	2007	PB/PE-B	AB623099	This study

BRbv1198	Cattle	Brejinho	PE	2007	PB/PE-B	AB623106	This study

BRbv1199	Cattle	Patos	PB	2008	PB/PE-B	AB623100	This study

BRbv1200	Cattle	Patos	PB	2008	PB/PE-B	AB623101	This study

BRbv1201	Cattle	Patos	PB	2008	PB/PE-B	AB623102	This study

BRbv1204	Cattle	Patos	PB	2008	PB/PE-B	AB623103	This study

BRbv1206	Cattle	Patos	PB	2008	PB/PE-B	AB623104	This study

BRbv1207	Cattle	Patos	PB	2009	PB/PE-B	AB623105	This study

BRbv1209	Cattle	Vitória de Santo Antão	PE	2008	PB/PE-B	AB623107	This study

BRbv1210	Cattle	Venturosa	PE	2008	PB/PE-A	AB623108	This study

BRbv1211	Cattle	Pedra	PE	2008	PB/PE-A	AB623109	This study

BRbv1212	Cattle	Venturosa	PE	2008	PB/PE-A	AB623110	This study

BRbv1213	Cattle	Garanhuns	PE	2008	PB/PE-A	AB623111	This study

BRbv1214	Cattle	Venturosa	PE	2008	PB/PE-A	AB623112	This study

BRbv1215	Cattle	Paranatama	PE	2008	PB/PE-B	AB623113	This study

BRbv1216	Cattle	Venturosa	PE	2008	PB/PE-A	AB623114	This study

BRbv1217	Cattle	Belo Jardim	PE	2008	PB/PE-B	AB623115	This study

BRbv1218	Cattle	Belo Jardim	PE	2009	PB/PE-B	AB623116	This study

BRbv1219	Cattle	Lajedo	PE	2009	PB/PE-B	AB623117	This study

BRbv1220	Cattle	Garanhuns	PE	2009	PB/PE-A	AB623118	This study

BRgt249(BR-Pgt1)	Goat	Patos	PB	2003	PB/PE-B	AB206437	[6]

BRgt1205	Goat	São Mamede	PB	2008	PB/PE-B	AB623077	This study

BRhr31	Horse	Ipora	GO	1998		AB083804	[17]

BRhr1196	Horse	Patos	PB	2007	PB/PE-B	AB623076	This study

BRsp250(BR-Psp1)	Sheep	Patos	PB	2003	PB/PE-B	AB206438	[6]

BRsp1171	Sheep	Santa Terezinha	PB	2004	PB/PE-B	AB623078	This study

BRsp1203	Sheep	Patos	PB	2008	PB/PE-B	AB623079	This study

*1 Names in parentheses are designations from Shoji *et al. *(2006)

*2 State abbreviations are as follows: *PB *Paraíba, *PE *Pernambuco, *GO *Goiás, *SP *São Paulo, *RJ *Rio de Janeiro, *MT *Mato Grosso, *TO *Tocantins, *MA *Maranhão, *PA *Pará, *MS *Mato Grosso do Sul

*3 Lineages are based on the phylogenetic tree of Figure 1

*4 We determined extra sequences in this study

## Competing interests

The authors declare that they have no competing interests.

## Authors' contributions

NM performed the molecular genetic studies and edited the manuscript; HK performed the RT-PCR and sequencing; TI, JABA, MLCRS, FHI, and TS participated in the study design, management, and coordination, and assisted in drafting the manuscript. All authors read and approved the final manuscript.
